# 

**Published:** 1914-05-16

**Authors:** 


### CONTENTS.

| | PAGE |
|---|---|
| **The Danger of a Short Sanatorium Course** ... | 169 |
| **The Rural Workhouse Infirmary...** ... ... | 170 |
| **Hospital and Institutional News** ... ... ... | 171 |

A Hospital President as Governor-General—Septic Throats in the Staff at Cambridge—The Budget and Pathological Research—The Government and Pathological Laboratories—Programme of the Newcastle Conference—Newcastle Conference: Second Day—Personalia at the Conference—A Well-known Secretary's Resignation—A Chairman on a Secretary's Work—The Ward Locker Competition: Who are the Culprits?—Specialist Staffs for Poor-Law Infirmaries—The Deadlock at Droitwich—The Asylum Workers' Association—Physician's Claim to Peerage Allowed—Do Cottage Hospitals get all the Legacies?—District Medical Officers and Operation Fees—The Salaries of Sanatorium Superintendents—The "Unhonoured" Status of Guardians—An Alternative to Combined Unions—Our Report and its Sequel at Guildford—A Well-Edited Annual Report—The Dearth of House Surgeons and How to Meet It—The Reporting of Inquests—A Lady Educationist on Medicine—The Pharmaceutical Council Election—This Week's Drug Market—To Our Readers.

[*See over.*]

### CONTENTS—(*continued*).

PAGE

**The Tendency of Medico-Psychology** ... ... 177

Freud's Theory and its Critics.

**Mental Hospitals and After-Care** ... ... 178

**The Facts of Syphilis on the Stage** ... ... 179

Plays by an English Doctor and a French Poet.

**Hospital Facts for an Interested Public** ... 181

**Royal Chest Hospital Dinner** ... ... ... 182

**Reports on Hospitals of the United Kingdom**— 183

By SIR HENRY BURDETT, K.C.B., K.C.V.O.

Margate Cottage Hospital.

Royal Sea Bathing Hospital, Margate.

Uckfield Cottage Hospital.

**Buildings Contemplated and in Progress** ... 184

**The History of St. George's Hospital** ... ... 185

Some Eighteenth Century Institutional Officers.

**Round the World** ... ... ... ... ... 186

Fellow-Workers and their Doings.

**The Editor's Letter-Box** ... ... ... ... 187

Should the Chairmanship go Round?—The Marriage of Faith and Science—What Keeps a Man off the Panel?—Heating by Gas—Premature Burial.

PAGE

**Doctors' Wills** ... ... ... ... ... ... 188
**The Department of Modern Nursing** ... ... 189
St. Bartholomew's Hospital.
**The London Clinical Congress** ... .. ... 191
**Practical Points in Hospital Mechanics** ... 192
The Control of Air Temperature by Thermostats.
**Legal Notes for Institutional Workers** ... 193
A Mental Specialist's Fees—Education Authorities and Medical Associations—Bequest to an Attendant—A Point for Public Health Officers.
**Personalia** ... ... ... ... ... ... 193
**The National Medical Union (Official)** ... ... 194
The London Organisation Committee.
**The Profession of Medicine** ... ... ... 196
One Hundred and Thirty Patients a Day!
**The Institutional Worker** ... .. (Suppl.) 1
Gas in Hospitals: Waste and its Cure.
Questions for May.
Questions Invited.

